# The first decade of antiretroviral therapy in Africa

**DOI:** 10.1186/1744-8603-7-33

**Published:** 2011-09-29

**Authors:** Nathan Ford, Alexandra Calmy, Edward J Mills

**Affiliations:** 1 Médecins Sans Frontières, Geneva, Switzerland; 2 Centre for Infectious Disease Epidemiology and Research, University of Cape Town, South Africa; 3 HIV/AIDS Unit, Infectious Disease Service, Geneva University Hospital, Switzerland; 4 Faculty of Health Sciences, University of Ottawa, Canada

## Abstract

The past decade has seen remarkable progress in increasing access to antiretroviral therapy in resource-limited settings. Early concerns about the cost and complexity of treatment were overcome thanks to the efforts of a global coalition of health providers, activists, academics, and people living with HIV/AIDS, who argued that every effort must be made to ensure access to essential care when millions of lives depended on it. The high cost of treatment was reduced through advocacy to promote access to generic drugs; care provision was simplified through a public health approach to treatment provision; the lack of human resources was overcome through task-shifting to support the provision of care by non-physicians; and access was expanded through the development of models of care that could work at the primary care level. The challenge for the next decade is to further increase access to treatment and support sustained care for those on treatment, while at the same time ensuring that the package of care is continuously improved such that all patients can benefit from the latest improvements in drug development, clinical science, and public health.

## Introduction

Since 2001, the international effort to scale up antiretroviral therapy (ART) in the developing world has been one of the most important programmes in global health [1]. Initially, there was considerable reluctance to provide ART in developing countries, due to concerns that treatment was too expensive, too complex, and that drug resistance would be promoted by inadequate programmes [2]. In particular, it was argued that ART was not cost-effective and that prevention interventions should be prioritized [3].

Despite these concerns, treatment programmes began to deliver ART at scale, and in less than a decade, more than five million people were successfully started on treatment. This remarkable progress was supported by a global coalition of doctors, patients, civil society actors, governments, and non-governmental organizations, who refused to accept that millions of people could be consigned to an early death from a disease that in developed countries had been transformed into a chronic, manageable condition.

This article provides an overview of the main policy and delivery challenges to the provision of effective ART in resource-limited settings, before outlining some of the future challenges for the coming years.

### Global advocacy to reduce the cost of treatment

The early reluctance to support ART for developing countries was driven by both public health caution and treatment cost. The fact that antiretroviral medicines were priced beyond the reach of most people who needed them in Africa had long been an international concern: at the International AIDS Conference in Stockholm in 1988 there was debate about how to ensure people in the developing world could access the treatment of that time - zidovudine monotherapy - which was marketed at a price of US$8000 per year [4]. Triple therapy, available in developed countries since late 1996, was considered far too expensive for resource-limited settings, and UN agencies [5], academics [3], and major donors alike [6] all argued against providing treatment in favour of focusing funding on prevention. As a consequence, many high-prevalence countries were slow to adopt national treatment plans.

Civil society groups, and in particular people living with HIV/AIDS, were crucial to breaking the deadlock. Patient groups in Thailand, Brazil, South Africa, India, Kenya, Uganda, and other high-burden countries formed alliances with health providers, non-governmental organizations, and health groups in developed countries to argue the case that the cost of treatment was too high [7]. Activist demonstrations took place across the world from New York to Bangkok to raise attention about the global inequities in access to treatment [8].

In 2002, a landmark legal case was to change the landscape. In South Africa, home to the largest number of people living with HIV/AIDS, the government fought (and arguably won) a court case against a consortium of 39 pharmaceutical companies over a law that would allow the government to source more affordable antiretrovirals from neighbouring countries [9].

Thailand and Brazil also played a critical part. Both countries established public capacity to produce medicines at a fraction of the price demanded by multinational pharmaceutical companies. These two countries played a leadership role by challenging the international monopolies of antiretroviral drugs and producing generic versions for a fraction of the price of the patented equivalents [10].

Widespread access to affordable antiretrovirals became feasible after the announcement by an Indian generics manufacturer in early 2001 that triple therapy could be manufactured for less than a dollar a day. This established a dynamic of global market competition that in 10 years has brought down the price of standard triple therapy from $US 10,000 per patient/year to almost $US50 [11]. Today, over 80% of ART used in low-income and middle-income countries is purchased from Indian generics companies [12]. The dramatic reduction in the cost of treatment was essential to shifting the cost-effectiveness equation, and from 2003 several international funding streams were established to support ART scale up, notably the Global Fund to Fight AIDS, Tuberculosis and Malaria and the US President's Emergency Plan for AIDS Relief [1].

### Overcoming the human resource crisis

As programmes began to enrol increasing numbers of patients, it became clear that the lack of qualified health personnel, particularly in Africa, would prove to be a major bottleneck in increasing access to treatment [13]. Whereas in high-income countries HIV/AIDS has traditionally been managed by a range of specialists from dermatology to oncology, health centres in sub-Saharan Africa faced with a dominant proportion of the global AIDS burden have a critical shortage of the most basic essential health staff. High HIV-prevalence countries like Malawi have 100 times fewer doctors per population than the USA [14].

A simplified treatment paradigm was required in resource-limited settings, entailing a shift from a specialized medical approach to a public health approach, in which the majority of clinical tasks would be undertaken by lower health cadres such as nurses. Given the vast numbers of lives being lost to HIV/AIDS every day, task-shifting strategies were initially employed outside of a formal evidence base. Rather than waiting for randomized trial data to show that nurses could perform as well as doctors in the prescribing of antiretrovirals, operational research was conducted to assess the effectiveness of such a strategy at the same time as it was being rolled out as national policy. Countries such as Lesotho [15], South Africa [16], and Malawi [17] demonstrated that with adequate training and supervision, routine clinical management of patients on ART could be delegated to nurses. The effectiveness of task shifting was subsequently confirmed by randomized trials [18], and substantial programmatic evidence has now accumulated around the benefits of task shifting in terms of increased access to care and improved team dynamics [19].

### Simplifying drug regimens and monitoring

The delivery of ART at the primary care level required a regimen that is easy to store, simple to take, and could be administered by lesser-trained health cadres via standardized guidelines. The development of fixed-dose combination ART was one answer to these requirements. World Health Organization (WHO) guidelines for antiretroviral therapy in resource-limited settings, first issued in 2001, recommended a regimen of nevirapine, stavudine, and lamivudine, as the preferred option [11]. This recommendation provided crucial scientific and political support for the use of a simple, affordable twice-daily regimen [20]. Implementation at large scale began in 2003, and by 2008, access to antiretroviral drugs in low-income and middle-income countries had risen tenfold [21] As well as providing guidance on drug regimens, the WHO guidelines also addressed the need for simplified toxicity and efficacy monitoring. The ability to perform CD4 counts and monitor viral load and levels of various markers of toxicity, although desirable, should not be a precondition to starting treatment.

### Decentralizing HIV/AIDS care to the primary care level

Task shifting and simplification strategies have been essential for supporting equitable access to care. Across sub-Saharan Africa, doctors are in short supply and for the most part are located in hospitals in cities: rural parts of South Africa for example have 14 times fewer doctors than the national average, whereas over half of Mozambique's doctors are working in the capital city, Maputo [14]. Because of this uneven distribution of clinical staff, policies that insist on doctor-based provision of antiretroviral therapy have been, by default, polices that limit access to treatment for populations living in rural areas.

Because distance to health services is associated with poorer adherence [22] and higher rates of defaulting from care [23], the decentralization of antiretroviral care to health centres in rural areas is critical for improving programme outcomes. Thus, another important modification to the standard model of HIV care practiced in high-income settings was the adaptation of services such that ART could be delivered effectively at the primary care level by health centre staff with supervision by clinical teams [16].

As the number of people on treatment continues to grow, there will be a need to go even further in the decentralization of care and develop models of chronic disease care outside of the formal health system. Studies from Uganda [24], Kenya [25] and Mozambique [26] have demonstrated that out-of-clinic approaches to ART management for stable patients are feasible, and this approach will become increasingly important in the future as a strategy to decongest overburdened health services and simplify treatment delivery.

### Improving quality of care

In the initial years of ART provision, HIV/AIDS was considered a humanitarian emergency, requiring a simple, rapid emergency response to reduce mortality as quickly as possible [27]. In order to provide effective, affordable care to the millions in need, adaptations to the Western model of care were required to simplify treatment regimens and adjust delivery models to the realities of resource-limited settings [28]. The need to continue to increase access to treatment for those not receiving it is still an urgent international priority. Recent evidence has also highlighted the need to treat people at an earlier stage during the course of their disease.

Data from European cohorts indicate that starting ART earlier (at CD4 350 cells/mm^3 ^or earlier) results in significant survival gains [29]; other cohort analyses from the USA also showed a survival gain by treating even earlier, at 500 CD4 cells/mm^3 ^[30]. The deleterious role of chronic, ongoing HIV replication is becoming clearer - and thus the risk of non-AIDS related complications such as cardiovascular diseases and non AIDS-defining cancers is a major contributor of the morbidity in HIV-infected individuals [31]. As a result, US, French, and European guidelines have recently been revised and recognize that treatment can be initiated as early as below 500 CD4 cells/mm^3^, especially in patients with other co-morbidities, aged over 50, or with organ dysfunction [32].

In line with this evidence, WHO revised its treatment guidelines for resource-limited settings at the end of 2009, recommending a move towards initiation at 350 cells/mm^3 ^[33] (previous WHO guidelines recommended treating patients at CD4 < 200 CD4 cells/mm^3^). However, treating earlier increases the number of people eligible for treatment, and donors and countries are still reluctant to support this policy shift.

Another challenge has been to ensure access to some of the newer drugs with better efficacy and side-effect profiles that are brought to market. The standard treatment regimen in developing countries has relied on stavudine, a drug that is relatively cheap (currently available as a combination costing less than US$60 per person per year), availability as a fixed-dose combination, good early tolerability, and its safety for use in pregnant women [11]. However, the higher rate of mitochondrial damage and toxicity associated with stavudine that have led its use to be progressively abandoned in developed countries [34]. In 2009, WHO revised its guidelines to recommend a move away from stavudine towards more drugs with a better safety profile, including tenofovir, which is also available as a once-daily regimen [35]. The relatively higher cost of this regimen has limited its inclusion in national protocols. Renewed advocacy efforts are needed to ensure that the price of tenofovir and companion drugs such as efavirenz comes down, that sufficient tenofovir production can be secured, and that promising new drugs in the development pipeline are made accessible at an affordable price as soon as they become available.

## Challenges for the next phase of antretroviral delivery

Ten years ago, global inaction against HIV/AIDS was labelled as a crime against humanity [2]. A growing international movement fought against the high cost of treatment and in just a few years succeeded in reducing the price of ART to a fraction of its original price [7]. Small pilot programmes that carefully selected a few dozen patients for treatment were rapidly swept away by demand and rapidly evolved into district wide programmes treating thousands of patients [36]. Treatment outcomes were evaluated and found to be as good as those reported in Western settings [37]. The model of ART care was adapted from a resource-intensive individualized approach to a public health programme that could be delivered by nurses at the clinic and community level [15]. Contrary to early fears, ART delivery was, after careful analysis, found to be supportive of health system strengthening [38].

As coverage of antiretroviral therapy increased, so the broader benefits of ART became apparent. In Malawi, adult mortality within the general population fell by a third as ART access increased [39], and similar declines in mortality have been reported elsewhere [40]. There is also emerging evidence to suggest that increased ART coverage may have an impact on prevention by reducing the population level viral load and thereby reducing the overall risk of transmission [41]. Models suggest that widespread ART coverage will result in a level of virological suppression at the population level that will reduce [42] or even eliminate [43] HIV transmission, and clinical trials have recently reported significant reductions in HIV incidence associated with earlier initiation of ART [44]. The preventive effect of antiretroviral therapy is currently greater than for other biomedical interventions such as microbicides [45], vaccines [46] or pre-exposure prophylaxis [47] to prevent HIV transmission through sexual contacts.

Enrolment and retention in care is an important challenge. In order to ensure sustained delivery of ART to increasing numbers and realize the potential preventive benefits of widespread treatment coverage, efforts are needed to reinforce the treatment cascade all along the pathway from HIV testing to early initiation to lifelong adherence to treatment. Recent reports indicate substantial rates of attrition at each step along the care pathway [48]. An important challenge for the next phase of ART scale up, therefore, is to identify and implement interventions to improve uptake and retention.

Despite these major advances, there is a sense that many of the important lessons of the past decade are being forgotten. In 2010, the high cost of treatment was again cited as a reason to accept sub-optimal care. The latest WHO guidelines recommend replacing older drugs long-abandoned in high-income countries with more durable and less toxic alternatives, but because these newer drugs are more expensive, developing countries are reluctant to make the change [11]. Just as the early benefits of ART were ignored in favour of cheaper interventions despite a clear mortality cost, this latest evidence is overlooked by donors who defend a policy of delaying treatment in order to ration resources [49]. This is shortsighted. Given that CD4 cells deplete at approximately 90 cells per year, the savings made by delaying initiation is around $300. But the difference in terms of long-term survival is substantial: a patient initiating therapy at the age of 20 with a CD4 count below 200 has an 8 year loss of life expectancy compared with initiation above 200 cells [50].

In 2005, the international community committed to a goal of achieving universal access to antiretroviral therapy by 2010. Not only have we failed to achieve that goal, but also the sustainability of gains made to date is under threat from multiple sides. Clinics are reporting major stock ruptures of antiretrovirals due in part to insufficiencies in Global Fund financing [51]. International advisors are suggesting that treatment numbers should simply be frozen. The concept of the "efficiency" of drug delivery is now the standard for programme evaluation.

A decade ago, those in the international community who did not support the scale up of ART in Africa could argue that it was untested. In 2011, it is now clear that treating HIV/AIDS on a large scale is entirely possible. Improving the basic package of care can limit side-effects and delay the need for patients to switch to more expensive second or even third-line regimens, whereas treating earlier will potentially yield massive public health benefits in terms of reduced transmission of HIV and other diseases.

The challenge for the next decade is to increase access to treatment and support sustained care for those on treatment, while at the same time ensuring that the package of care is continuously improved such that all patients - whether they happen to be born in the developed world or the developing world - can benefit from the latest improvements in drug development, clinical science, and public health.

## Competing interests

The authors declare that they have no competing interests.

## Authors' contributions

NF conceived of the study and wrote the first draft. All authors contributed to subsequent drafts. All authors have read and approved the final manuscript.
